# Schwannoma of the colon: A case report

**DOI:** 10.3892/ol.2014.2545

**Published:** 2014-09-17

**Authors:** WALTER BUGIANTELLA, FABIO RONDELLI, LORENZO MARIANI, LUCINA PEPPOLONI, ENRICO CRISTALLINI, ENRICO MARIANI

**Affiliations:** 1 General Surgery, ‘San Giovanni Battista Hospital’, USL Umbria 2, Foligno I-06034, Italy; 2 Department of Surgery, University of Perugia, Perugia I-06132, Italy; 3 Pathological Anatomy, ‘San Giovanni Battista Hospital’, USL Umbria 2, Foligno I-06034, Italy

**Keywords:** immunohistochemistry, schwannoma, colon, tumor, surgery

## Abstract

Schwannomas are rare tumors originating from the Schwann cells, which form the neural sheath. These tumors occur most frequently in the head, neck, arms and limbs. Primary schwannomas of the colon and rectum are extremely rare; they are usually benign, but in extremely rare cases (2%), they can present with malignant degeneration if not surgically removed. The current study presents the case of a 65-year-old male with blood in the feces who underwent a colonoscopy that revealed an oval-shaped mass covered by ulcerated mucosa. A standard biopsy examination indicated a gelatinous carcinoma, and the patient consequently underwent a laparoscopic resection of the left colon. Histological examination revealed a schwannoma. Immunohistochemistry showed the tumor to be positive for S100 and vimentin, but negative for cluster of differentiation (CD)117, cytokeratin (CK)7, CK20, chromogranin, actin and synaptophysin, with a Ki-67 proliferative index of 3%. Lymph nodes were not involved. Overall, pre-operative biopsy examinations may be difficult for schwannomas, and immunohistochemistry is necessary for the correct diagnosis of this condition. In contrast to gastrointestinal stromal tumors, schwannomas are negative for CD117 and positive for S100 protein and vimentin. A Ki-67 index of ≥5% is strictly correlated with greater tumor aggressiveness. Therefore, the gold standard treatment for schwannomas is oncological radical surgical resection.

## Introduction

Schwannomas are rare tumors histologically derived from Schwann cells that form the neural sheath. The tumors occur most frequently in the head, neck, arms and limbs. Primary schwannomas of the colon and rectum that are not associated with von Recklinghausen’s disease are extremely rare (2–6% of all stromal tumors of digestive tract); only ~50 cases are reported in the literature worldwide (1). Schwannomas are mostly asymptomatic, but can present with non-specific symptoms, including pain, fatigue and fever. Occasionally, rectal bleeding and signs of colonic obstruction may also occur (2). The majority of primary schwannomas are benign and asymptomatic; nevertheless, the possibility of malignant degeneration exists and is directly associated with the tumor dimensions. Radical excision with margins free of disease is the treatment of choice, since the response to chemotherapy and radiotherapy remains uncertain (3). Despite aggressive surgical management, these tumors may appear with a high rate of local recurrence and malignant degeneration. In such cases, there are few treatment options and the prognosis is poor (3).

## Case report

A 65-year-old male underwent colonoscopy due to the presence of occult blood in the feces. The procedure revealed an oval-shaped mass of roughly 2.5 cm in diameter covered by ulcerated mucosa, located 22 cm from the anal verge. Endoscopic ultrasonography revealed an hypo-oechogenic, homogeneous lesion, with well-defined margins, originating from the submucosa. A standard biopsy examination indicated a gelatinous carcinoma. A total body computed tomography scan excluded metastasis, therefore the patient underwent a laparoscopic resection of the left colon.

**Figure 1:**
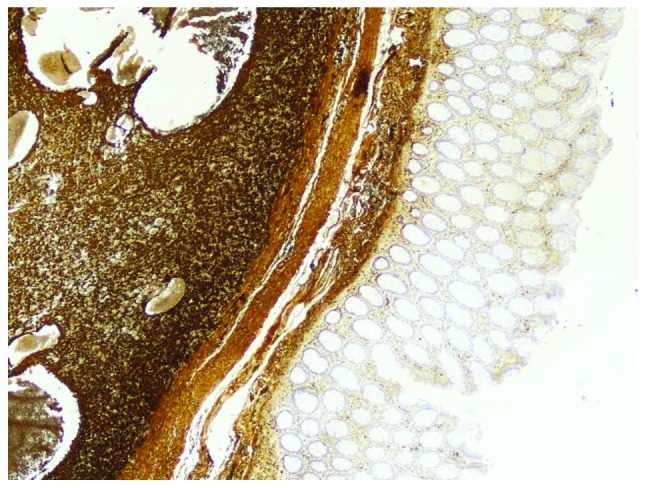
Immunohistochemistry of the colonic neoplasm showing S100 positivity.

A histological examination revealed a schwannoma covered by an ulcerated mucosa. Immunohistochemistry showed that the tumor was positive for S100 and vimentin, but negative for cluster of differentiation (CD)117, cytokeratin (CK)7, CK20, chromogranin, actin and synaptophysin, with a Ki-67 proliferative index of 3% (Fig. 1). The lymph nodes were not involved. The patient did not undergo any adjuvant therapy. A total-body computed tomography scan was performed every six months and a colonscopy was performed yearly. No local or distal recurrence of the lesion was identified following two years of follow-up. Written informed consent was obtained from the patient for publication of this study.

## Discussion

Schwannomas are rare tumors of ectodermic origin growing from the neural sheath (2). In the digestive tract they arise from the autonomous nervous system; more frequently from Auerbach’s plexus, and less frequently from Meissner’s plexus. The tumors are usually benign, slowly-growing neoplasms, although in extremely rare cases they can present with malignant degeneration if not surgically removed (1–3). The incidence rate is the identical for males and females, and increases between the sixth and seventh decades of life. The stomach and the small bowel are the most frequently involved sections of the digestive tract (83 and 12%, respectively) (1).

Macroscopically, schwannomas are lobulated and well-delimited tumors, often with a cystic pattern.

Neoplasms originating from Auerbach’s plexus protrude into the intestinal lumen and are characterized by a non-pedunculated oval-shaped mass, whereas those arising from Meissner’s plexus are often similar to pedunculated polyps (4).

Schwannomas are histologically characterized by Verocay corpuscles, a lymphoid cuff and a spiral-like form consisting of densely arrayed spindle-shaped cells, palisade arrangements and loose reticular networks of cells. Two histological growth patterns have been described: Antoni A (dense growth of fusiform cells compactly arranged in palisades to form Verocay bodies) and Antoni B (the fusiform cells are more loosely distributed with rounded or elongated nuclei, with a great quantity of myxoid stroma and xanthomatous histiocytes) (1,4). Schwann cells covering a basal membrane are not generally identified, however, there may be undifferentiated mesenchymal cells, smooth muscle cells and cells with neural characteristics or with mixed differentiation of the neural/muscle type (1).

Although certain studies consider schwannomas to be a subtype of gastrointestinal stromal tumor (GIST) belonging to the gastrointestinal autonomic nerve tumors (GANT), there are significant histopathological and immunohistochemical differences (2,3). In contrast to GISTs, schwannomas are consistently negative for CD117 (KIT) and usually negative for CD34, CKs, smooth muscle actin and desmin, but strongly positive for S100 protein and vimentin. (1,4,5). Moreover a lymphoid cuff, diffuse lymphoid infiltration, an impression of cellular heterogeneity, focal nuclear atypia and a microtrabecular pattern are not features of GISTs (6).

The precise biological behavior of schwannomas is not fully understood, mostly due to their rarity. Although they are considered benign neoplasms, possible malignant behavior must not be ignored. In fact, Das Gupta and Brasfield reported that in 2% of cases, distant metastasis may be observed (7). A correct histological diagnosis requires investigation of the Ki-67 proliferative index (MIB-1), as its positivity (≥5%) is strictly correlated with greater tumor aggressiveness. A mitotic activity rate of more than five mitoses per field at high magnification and a tumor size >5 cm tend to be associated with a high risk of metastasis and recurrence. A low rate of mitosis and the absence of atypical mitotic figures and nuclear hyperpigmentation characterize a benign lesion (1).

Pre-operative biopsy examinations may be difficult, and immunohistochemistry is necessary for the correct diagnosis of a schwannoma. Currently, the gold standard treatment for schwannomas is complete surgical resection with oncological radical intent. Our experience leads us to suggest that, even if the lymph nodes are not involved, radical surgery is the best treatment.

## Abbreviations

GIST: gastrointestinal stromal tumor
GANT: gastrointestinal autonomic nerve tumor
